# Exposure to Music Alters Cell Viability and Cell Motility of Human Nonauditory Cells in Culture

**DOI:** 10.1155/2016/6849473

**Published:** 2016-07-12

**Authors:** Nathalia R. Lestard, Marcia A. M. Capella

**Affiliations:** Institute of Biophysics Carlos Chagas Filho, Federal University of Rio de Janeiro, 21941-902 Rio de Janeiro, RJ, Brazil

## Abstract

Although music is part of virtually all cultures in the world, little is known about how it affects us. Since the beginning of this century several studies suggested that the response to music, and to sound in general, is complex and might not be exclusively due to emotion, given that cell types other than auditory hair cells can also directly react to audible sound. The present study was designed to better understand the direct effects of acoustic vibrations, in the form of music, in human cells in culture. Our results suggest that the mechanisms of cell growth arrest and/or cell death induced by acoustic vibrations are similar for auditory and nonauditory cells.

## 1. Introduction

Despite being an integral part of virtually all cultures in the world, little is known about how music affects us. A number of studies suggest that music may be useful in medical care, alleviating stress and nociception in patients undergoing surgical procedures, as well as in cancer and burned patients [1–6] but the mechanisms by which these effects occur are still unidentified. It is commonly accepted that the effects of music are secondary to emotional responses, but Møller and Pedersen affirmed that vibrotactile sensations and a feeling of pressure might also occur in the chest and throat while hearing sounds [7].

Since the beginning of this century several studies suggested that the response to music, and to sound in general, is complex and might not be exclusively due to emotion, given that cell types other than auditory hair cells can also directly react to audible sound. For example, it has been shown that sound wave stimulation makes significant changes to protein structure of tobacco cells, producing an increase in *α* helix and a decrease in *β* turn [8]; also, sound stimulation produced effects on the cell cycle of* Chrysanthemum* [9] and in the callus growth of* Dendranthema morifolium* [10]. More recently, it was demonstrated that the tonal sounds of 1 kHz and 5 kHz promoted the growth of* Escherichia coli* [11, 12].

When considering mammalian cells, an increase in corticosterone serum levels after environmental noise exposure and a long-term reduction of proliferating cells in the hippocampal of noise exposed rats were observed, suggesting that exposure to chronic environmental noise at young ages produces persistent impairment to nonauditory cells, altering cell proliferation in the hippocampal formation [13]. It has also been shown that a frequency of 261 Hz was able to alter the growth of human gingival fibroblasts in culture [14] and recently we showed that music (and not only pure frequencies) can lead to several effects in human cells in culture, altering cell cycle, proliferation, viability, and binding of hormone [15]. Since music is a summation of several sound frequencies, and since sound is indeed a mechanical vibration, which can cause mechanical stress, it seems not odd that music can cause direct effects in mammalian cells. The present report was thus designed to better understand the direct effects of acoustic vibrations in the form of music in human cells in culture.

## 2. Material and Methods

### 2.1. Cells

MCF-7 and MDA-MB-231 are human breast cancer cell lines with characteristics of epithelial cells. The cells were grown in Dulbecco's Modified Eagle Medium with penicillin and streptomycin supplemented with 10% fetal bovine serum (all from Invitrogen, Brazil) in disposable plastic bottles (Techno Plastic Product, Germany), at 37°C until confluence. For each experiment, cells were platted on 40 mm plastic Petri dishes (TPP, Germany), at 1 × 10^5^ cells/dish. For cell migration experiments 5 × 10^4^ cells/dish were platted on each well. The experiments were performed 24 h after seeding, to ensure uniform attachment of the cells. Each experiment was repeated at least 3 times.

### 2.2. Treatment with Music

The cells were exposed for 30 min to one of the three compositions: Mozart's Sonata for Two Pianos in D major, KV. 448, first movement; Beethoven's 5th Symphony, first movement; Ligeti's Atmospheres, first movement; at 37°C in an incubator chamber. The cells were exposed to the music using a coaxial speaker, model ar 5c, ref. 83503, 60 Watts, from UB Natts Eletroacústica (São Paulo, Brazil), placed at the roof of the incubator chamber, whose walls were lined with cork and foam. As controls, the cells were exposed to silence (speakers were turned off in the incubator) or to the speakers plugged to energy without any sound produced, to observe a possible action of the background noise or the magnetic field produced by the speakers. Since Beethoven's Fifth and Ligeti's Atmospheres move continually from piano to forte and vice versa, there is no way to imprint a constant sound pressure. Therefore, the sound pressure levels were maintained between 70 and 100 dB to all compositions.

### 2.3. Apoptosis Assay

After exposure to music the cells were incubated for 24, 48, and 72 h. At each time, the supernatants of cells cultures were collected into conical tubes (in order to collect suspension cells) and the remained attached cells were washed 2 times with phosphate buffered saline (PBS), harvested with trypsin, and collected into the same conical tube. The tubes were centrifuged 5 min (650 ×g), the supernatant was discarded, and the pellet was resuspended in 100 *μ*L of annexin V binding buffer (Invitrogen, Brazil) homogenized and transferred to flow cytometry tubes. The cells were then stained with 0.5 *μ*L annexin V-FITC and 1 *μ*L propidium iodide (PI) (100 *μ*g/mL) for 20 min in the dark. After this time 200 *μ*L of annexin binding buffer was added and homogenized and the cells were analyzed in a FACScan flow cytometer (Becton and Dickinson, USA).

### 2.4. Signaling Pathway to Apoptosis

MCF-7 cells were cultured and exposed to Ligeti's Atmospheres as described above. After 48 h of incubation, the cells were collected and the levels of p53, phospho-p53, Bad, phospho-Bad, Cleaved Caspase 3, and Cleaved PARP were measured by ELISA with commercially available kits (PathScan Apoptosis Multi-Target Sandwich ELISA Kit #7105, CST Inc., USA). Absorbance was read at 450 nm.

### 2.5. Cell Migration

Cell migration was characterized using Transwell system 8 *μ*m pore size (Corning, USA). The cells were grown until 80% confluence, washed 2 times with PBS, and incubated with serum-free medium supplemented with 0.3% BSA. After 24 h starvation cells were washed 2 times with PBS, harvested with trypsin, and collected into conical tubes. After centrifugation for 5 min (650 ×g), the supernatant was discarded and the pellet was resuspended in serum-free medium at a density of 5 × 10^5^ cells/mL. An aliquot of 100 *μ*L of the cell suspension was added into the upper chamber of the Transwell system and 600 *μ*L of medium with 5% SFB was added into the lower chamber. The cells were randomly exposed for 30 min to the compositions and then incubated for 4 hours. The inserts were fixed in 1% paraformaldehyde for 30 min and stained with 1% crystal violet for 10 min. The inserts were then washed and the upper surface of the membranes was wiped with a cotton swab to remove nonmigratory cells. Migrated cells were counted in five randomly selected fields. The results are presented as mean cell numbers for membrane.

### 2.6. Statistical Analysis

Each experiment was repeated at least 3 times. Data are expressed as means ± standard error of the mean and were analyzed using Student's* t*-test or one-way ANOVA with Dunnett post test for comparison of the differences. Values of *p* less than 0.05 were considered statistically significant.

## 3. Results

**Figure 1:**
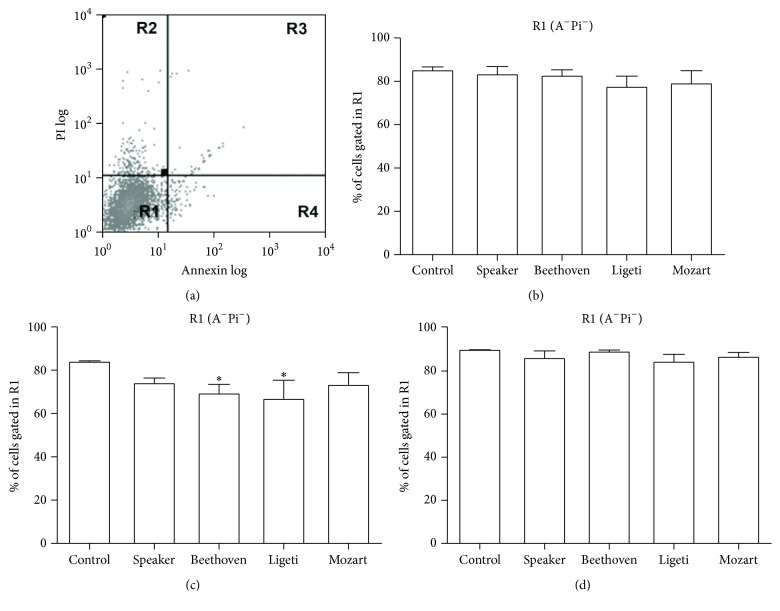
Apoptosis induced by music in MCF-7 cells. The cells were exposed to each composition as explained in Section 2 and incubated for 24, 48, or 72 h. Apoptotic cells were analyzed by flow cytometry with propidium iodide (PI) and annexin V-FITC staining. (a) Dot-plot of control cells, showing the regions used in Figures 1 and 2. (b) Percentage of cells gated in region R1 (live cells) 24 h after exposure to the compositions. (c) Percentage of cells gated in region R1 48 h after exposure to the compositions. (d) Percentage of cells gated in R1 72 h after exposure to the compositions. Data are presented as means ± SE of four independent experiments. ^*∗*^ *p* < 0.05 (significant when compared with control).

In a previous study we observed that the human breast cancer cell line MCF-7 responded to music by altering cell cycle and diminishing its viability [15]. However, the method used, Trypan blue assay, only permits visualization of dead cells, which lost membrane integrity. That method could not distinguish between necrotic and late apoptotic cells. It was of interest to know whether cell death was due to apoptosis, which is a more physiological type of cell death, or necrosis, which would indicate an abrupt, sudden, death. Therefore, we now used the annexin-PI assay. According to Figure 1, living cells are gated in region R1 of Figure 1(a), showing unlabeled cells (A^−^Pi^−^); cells undergoing apoptosis are gated in region R4 of Figure 1(a), which shows cells labeled only with annexin V (A^+^Pi^−^); and late apoptotic, dead cells, labeled with both annexin V and PI (A^+^Pi^+^), are gated in region R3 of Figure 1(a).

In Figure 1(c) it is shown that the percentage of live cells (R1) is significantly diminished 48 h after acoustic exposure to Beethoven's 5th Symphony or Ligeti's Atmospheres. The speaker alone and Mozart's composition did not reduce significantly the cell viability. Moreover, no significant difference was observed 24 h or 72 h after music exposition (Figures 1(b) and 1(d), resp.).

**Figure 2:**
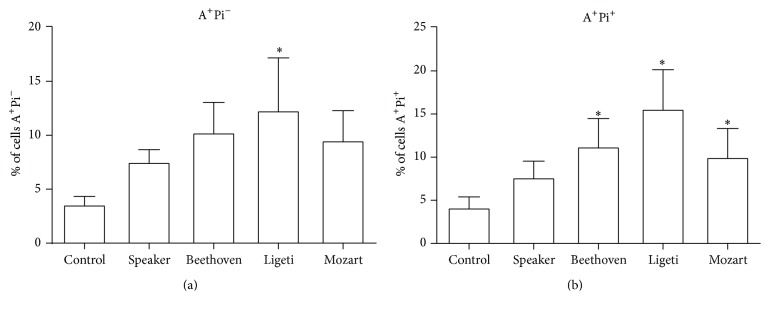
Apoptosis induced by music in MCF-7 cells. The cells were exposed to each composition and incubated for 48 h. (a) % of cells gated in region R4 of Figure 1(a) (positive for annexin V only). (b) % of cells gated in region R3 of Figure 1(a) (positive for annexin V and PI). Data are presented as means ± SE of four independent experiments. ^*∗*^ *p* < 0.05 (significant when compared with control).

In Figure 2 it is shown that Ligeti's composition increased significantly the percentage of apoptotic cells (region R4, A^+^Pi^−^) 48 h after acoustic treatment. Beethoven's and Mozart's compositions, as well as the speaker alone, have a tendency to increase the percentage of apoptotic cells, although the differences were not statistically significant in relation to the control. In addition, the three compositions increased significantly the percentage of dead cells (late apoptotic cells), gated in region R3 (A^+^Pi^+^).

**Figure 3:**
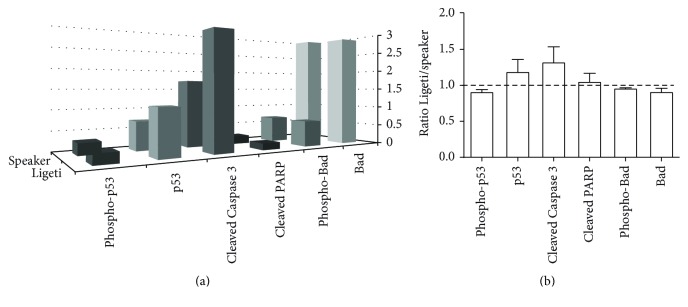
Treatment of MCF-7 cells with Ligeti's Atmospheres induced phosphorylation of p53 at Ser15, as well as cleavage of Caspase 3 as detected by PathScan® Apoptosis Multi-Target Sandwich ELISA Kit #7105. The cells were exposed to the composition or to the speaker alone and incubated for 48 h and the lysates were assayed as explained in Section 2. The absorbance readings at 450 nm are shown as a 3-dimensional representation in (a), while the ratio between Ligeti's Atmospheres and speaker is shown in (b).

Since these results suggest that cells are dying by apoptosis, we tried to identify the possible apoptosis pathway. To do this, we used Ligeti's Atmospheres, since it was the composition that led to the major increase in apoptotic cells (both A^+^Pi^−^ and A^+^Pi^+^ regions). The PathScan Apoptosis Multi-Target Sandwich ELISA Kit #7105 was used, which detects endogenous levels of p53 protein, phospho-p53 protein, Bad, phospho-Bad, Cleaved Caspase 3, and Cleaved PARP, key molecules in signaling pathways controlling survival and apoptosis. In Figure 3 a representative result from one experiment (Figure 3(a)) and the ratio between cells exposed to Ligeti's Atmospheres and cells exposed to speaker (Figure 3(b)) are shown. It can be seen that only phosphorylated p53 and Cleaved Caspase 3 were increased in cells exposed to Ligeti's Atmospheres, in relation to the speaker alone.

**Figure 4:**
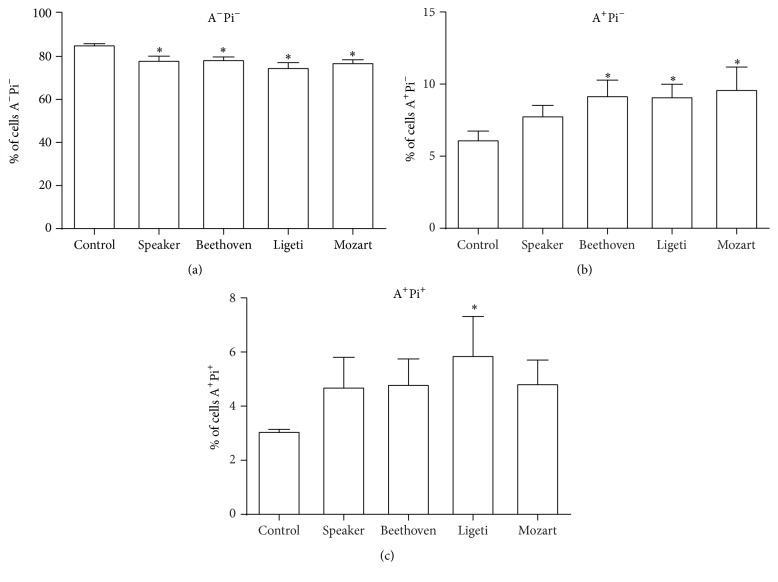
Apoptosis induced by music in MDA-MB-231 cells. The cells were exposed to each composition and incubated for 24 h. (a) % of cells gated in region R1 of Figure 1(a) (negative for both annexin V and PI). (b) % of cells gated in region R4 of Figure 1(a) (positive for annexin V only). (c) % of cells gated in region R3 of Figure 1(a) (positive for annexin V and PI). Data are presented as means ± SE of four independent experiments. ^*∗*^ *p* < 0.05 (significant when compared with control).

Because until now only one cell line was studied, we tested whether the effects observed so far could be observed in other cell lines: MDCK, a canine renal cell line with characteristics of distal nephron [16]; K562 and K562-Lucena, two human erythroleukemia cell lines, the latest being multidrug resistant [17]; and MDA-MB-231, a human breast cancer cell line previously used by our group that do not respond to estrogens [18]. The first three cell lines did not alter their viability after exposure to any compositions used (data not shown). However, the breast cancer cells MDA-MB-231 have also undergone apoptosis after exposure to music, but their response was different from that observed for MCF-7 cells, as can be seen in Figure 4.

In this figure it is shown that the three compositions, as well as the white noise of the speaker, reduced the percentage of viable cells (Figure 4(a)). Correspondingly, they increased the number of cells undergoing both early and late apoptosis.

**Figure 5:**
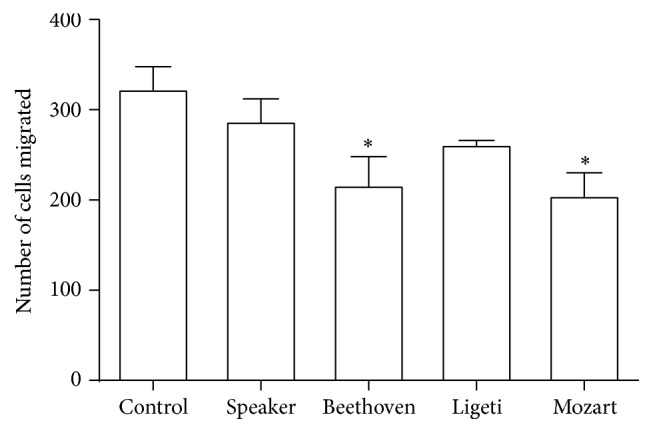
Exposition to music reduced the migration of MDA-MB-231 cells* in vitro*. Cells were exposed to each one of the three compositions, as explained in Section 2, and the cell migration was observed. ^*∗*^ *p* < 0.05 (significant when compared with control).

Since this cell line is potentially metastatic, we tried to evaluate whether music could alter this potential. For this, after exposure to music or speaker alone the cells were submitted to the cell migration assay, as described in Section 2. In Figure 5 it is shown that both Beethoven's 5th Symphony and Mozart's sonata significantly diminished the migration of MDA-MB-231 cells.

## 4. Discussion

In a previous study we showed that music could act directly on MCF-7, a human breast cancer cell line, altering cell cycle, proliferation, and viability. In the present report we extend our studies, trying to understand such effects and evaluating whether they could be observed in other cell types. Interestingly, although we tested four cell lines, we noticed that only the breast cancer cell line MBA-MD-231 reacted to music and that such reaction was different from that previously observed for MCF-7 cells. Possible explanations for this fact could be that the human erythroleukemia cell lines, being from blood origin, retain characteristics that help them to cope with mechanical stress. The same argument may be plausible for MDCK cells: being from distal nephron origin, they are familiar with mechanical stress due to tubular flow. Therefore, it is possible that the absence of response of these cell lines was related to their origins. This hypothesis takes into account that* in vitro* direct effects of music in nonauditory cells are related to mechanical stress, which is reasonable, since music is, after all, a mechanical vibration, which can cause mechanical stress. However, at present it is not possible to say whether such stress occurs externally (in the culture medium), inside cells themselves or both.

We observed that the two breast cancer cell lines reacted to music in different ways. While Mozart's composition did not alter cell viability of MCF-7 cells, it led MDA-MD-231 to apoptosis. Moreover, the speaker alone was able to significantly reduce MDA-MD-231 viability, while it had no effect on MCF-7 cells. Moreover, the compositions of Beethoven and Mozart inhibited MDA-MB-231 migration, without altering cell viability. These results suggest that the cellular response to music, and perhaps to sound in general, is dependent not only on the nature of the sound, but also on intrinsic characteristics of the cellular type. Since this is the second report about the direct effects of music in nonauditory cells in culture, more studies are needed to achieve comprehension of these phenomena.

In the present report we also tried to observe the pathway of apoptosis elicited by Ligeti's music in MCF-7 cells. By using an ELISA kit, we tested the key apoptotic molecules p53 protein, phospho-p53 protein, Bad, phospho-Bad, Cleaved Caspase 3, and Cleaved PARP and observed that only p53 and Caspase 3 were increased.

The p53 protein is associated with DNA repair, growth arrest, and apoptosis [19]. It is known that, after a DNA damage, p53 upregulates Bad transcription and that dephosphorylated Bad can heterodimerize with p53 and translocate to mitochondria [20]. Moreover, PARP-1 is also activated after DNA damage and it has been shown that the interplay between PARP-1 and p53 is dependent on the type of damage induced to DNA [21]. Since there was no alteration on both PARP-1 and Bad status after exposure of MCF-7 cells to music, it seems that the apoptosis induced by Ligeti's Atmospheres is not due to DNA damage, which is very reasonable, given that we do not expect that music will lead to DNA damage. We have previously shown that Ligeti's Atmospheres induced MCF-7 cells growth arrest in S-phase and also an increase in sub-G0 phase, which is in accordance to our present results. Therefore, taken together, our results suggest that exposure of MCF-7 cells to music can induce cell growth arrest and/or apoptosis. Interestingly, it has been shown that intense noise leads to hair cells apoptosis by a pathway dependent on p53 and Caspase 3 [22, 23]. Therefore, our results suggest that the mechanisms of cell death or growth arrest induced by sound (including music) are similar for auditory and nonauditory cells.

Finally, in the present study we evaluated cell fate within 3 days of experimentation. Longer observations for follow-up would bring other valuable data and could be interesting to understand the properties observed by music intervention to people with disease. We expect to do this in the future.
